# Edge Effects along a Seagrass Margin Result in an Increased Grazing Risk on *Posidonia australis* Transplants

**DOI:** 10.1371/journal.pone.0137778

**Published:** 2015-10-14

**Authors:** John Statton, Samuel Gustin-Craig, Kingsley W. Dixon, Gary A. Kendrick

**Affiliations:** 1 School of Plant Biology and UWA Oceans Institute, Faculty of Natural and Agricultural Science, University of Western Australia, Crawley, Western Australia; 2 Kings Park and Botanic Garden, Biodiversity and Conservation Building, West Perth, Western Australia; 3 Department of Environment and Agriculture, Curtin University, Bentley, Perth, Western Australia; Dauphin Island Sea Lab, UNITED STATES

## Abstract

A key issue in habitat restoration are the changes in ecological processes that occur when fragments of habitat are lost, resulting in the persistence of habitat-degraded margins. Margins often create or enhance opportunities for negative plant-herbivore interactions, preventing natural or assisted re-establishment of native vegetation into the degraded area. However, at some distance from the habitat margin these negative interactions may relax. Here, we posit that the intensity of species interactions in a fragmented *Posidonia australis* seagrass meadow may be spatially dependent on proximity to the seagrass habitat edge, whereby the risk of grazing is high and the probability of survival of seagrass transplants is low. To test this, transplants were planted 2 m within the meadow, on the meadow edge at 0m, and at 2m, 10m, 30m, 50m and 100m distance from the edge of the seagrass meadow into the unvegetated sand sheet. There was an enhanced grazing risk 0-10m from the edge, but decreased sharply with increasing distances (>30m). Yet, the risk of grazing was minimal inside the seagrass meadow, indicating that grazers may use the seagrass meadow for refuge but are not actively grazing within it. The relationship between short-term herbivory risk and long-term survival was not straightforward, suggesting that other environmental filters are also affecting survival of *P*. *australis* transplants within the study area. We found that daily probability of herbivory was predictable and operating over a small spatial scale at the edge of a large, intact seagrass meadow. These findings highlight the risk from herbivory can be high, and a potential contributing factor to seagrass establishment in restoration programs.

## Introduction

Habitat fragmentation through degradation or natural losses often results in the development of habitat edges or margins. When edges dissect a natural habitat and the opposing area is degraded, the natural habitat can be negatively impacted for some distance in from the edge [1]. This ‘edge effect’ develops along the margins of opposing habitats because of modified ecological conditions such as changes in micro-climate (e.g. light, temperature, soil moisture content, and humidity), nutrient availability, pollination and reproduction, species invasions, and predator-prey interactions [1]. Altered patterns of plant-herbivore interactions along vegetation edges are an additional, more imposing threat to natural recolonisation and restoration [2], but have not been investigated in seagrasses. Seagrass meadows have become increasingly fragmented from human disturbances [3], with resultant increase in ecological anomalies such as meadow margins. For plant conservation and restoration efforts, the emerging dominance of edge effects is an ongoing concern in both marine and terrestrial ecosystems [1,4].

Edge-facilitated herbivory can pose a significant constraint on the natural regeneration or restoration of terrestrial and marine plant communities. For example, browsing animals (e.g. deer, voles, mice) from adjacent forests selectively graze in nearby grassland reclaimed from surface mines or old fields, retarding woody plant colonization and additionally affecting species composition [5–7]. As with herbivores along terrestrial forest edges, patterns of herbivory and herbivore abundance in marine settings are also altered along habitat edges. Intertidal grazers target their feeding to edges of mussel beds, retreating into the mussel beds for safety [8]. This spatially-restricted, risk-averse foraging contributes to ‘browse zone’ patterns in which algae are entirely removed from the edges of mussel gaps but gap interiors remain largely ungrazed [9]. Similarly, in subtidal marine ecosystems, seagrass grazing ‘halos’ form along the edges of patch reefs [10]. Patch reefs act as aggregating structures for marine fauna, in particular, herbivorous fish and sea urchins use patch reefs as a refuge, venturing a distance beyond the reef to graze on adjacent seagrass meadows or transplants [11–14].

In naturally fragmented seagrass meadows, the occurrence of large, intact seagrass meadows may be analogous to patch reefs. For example, in a recent study in the eastern gulf of Shark Bay, Western Australia, [15] found that in bare sandy areas adjacent to nearby *Posidonia australis* and *Amphibolis antarctica* meadows, herbivorous fish (e.g. striped trumpeter—*Pelates octolineatus*) grazed upon seagrass leaves from a range of seagrass species that included the meadow forming *P*. *australis* and *A*. *antarctica*. [15] suggested that large seagrass species like *P*. *australis* and *A*. *antarctica* may act as refugia for foraging herbivorous fish that venture out to graze on seagrass in nearby and less structurally complex areas. However, the role of vegetated edges in regulating seagrass-herbivore interactions within fragmented seagrass meadows remains unclear.

Seagrass edges can modify habitat use by fauna. For example, both fish and invertebrate communities change with distance from the seagrass edge [16–19], and foraging patterns can also vary in response to the presence of seagrass edges [20–22]. Consequently, there is greater potential for indirect edge effects on seagrass structure and function through altered spatial patterns of predation or herbivory [23] though these interactions are not well understood for seagrasses.

Transplanting shoots or runners (rhizome divisions) of seagrass is the main method used to restore seagrass in degraded ecosystems [24], though success has been sporadic [25,26]. Restoration outcomes depend on the characteristics of the degraded site and its appropriateness for seagrass establishment [24,26,27]. Some degraded areas may not be able to be restored, owing to altered biotic and abiotic conditions [28], that are often ignored in restoration projects [29], but can make a degraded system or area resistant to restoration [28]. A clear understanding of these environmental factors militating against restoration is a critical step in providing the intervention approaches to deliver more effective restoration outcomes.

Because plant-herbivore interactions can be modified by distance from habitat edges, determining how far unfavourable edge effects permeate into a degraded habitat could provide a spatial context for restoration strategies in these environments. Here, we test whether the intensity of plant-herbivore interactions, in a fragmented seagrass meadow is spatially dependent on proximity to the remnant seagrass edge. Based on observations of seagrass transplant losses we hypothesize that a strong plant-herbivore-grazing interaction occurs within the unvegetated sand sheet near (within m’s) to the edge of the *Posidonia australis* meadow, and the probability of this interaction decreases with increasing distance (10’s of m) from meadow edge into the unvegetated sand sheet (up to 100m). Because *P*. *australis* is a large, habitat forming meadow and potentially acts as refuge for forging fish and other fauna, we expect to observe negligible grazing within the *Posidonia* meadow. We then investigate the patterns of survival after six months and propose mechanisms potentially influencing these patterns of survival.

## MaterialS and Methods

### Study Area

**Fig 1:**
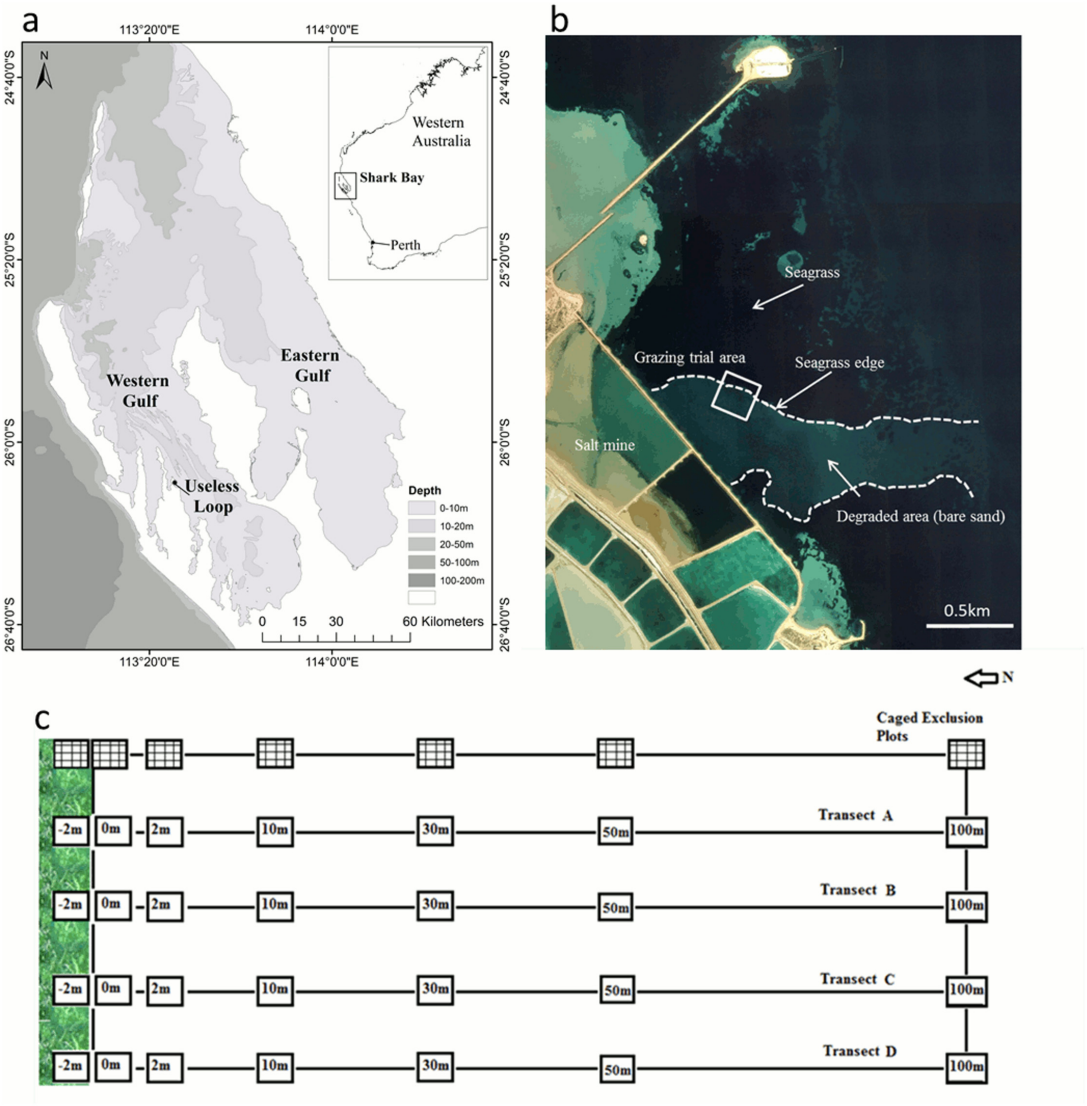
Map of Useless Loop, Shark Bay (a). Twenty five years after bitterns released from a nearby solar salt facility affected 120 ha of seagrass—dotted line indicates approximate edge of the degraded area (b). Grazing trial area (white box) located on the northern edge of the degraded seagrass habitat. Experimental design of grazing trial (c).

The World Heritage Area of Shark Bay is a shallow (< 15m) subtropical embayment located ca. 900 km north of Perth, Western Australia, with extensive seagrass communities considered one of the outstanding natural values for which the area was heritage listed [30]. Shark Bay contains 12 species of seagrass from temperate and tropical realms, covering an estimated 4500km^2^ [31]. *Posidonia australis* is among the most widespread species within the bay, covering ca. 200km^2^ [31]. This study was conducted at Useless Loop, in the western gulf of Shark Bay (Fig 1a). Useless Loop has been impacted by historical industrial activities, and has lost more than 120 ha of seagrass habitat, with little natural recovery, even after a prolonged period (ca. 25 years, [32]). The impacted area is characterized by a sheet of unvegetated sand surrounded by well-defined meadows of *Posidonia australis* nearshore, transitioning to *Amphibolis antarctica* meadows offshore (Fig 1b).

### Study Design

Herbivory assays on *P*. *australis* transplants were conducted over six days in June 2012 and a longer term (six months) assessment of transplant survival and establishment was conducted from beginning of June 2012 until end of November 2012. Fish surveys were conducted twice over six days in June 2012 and again in November 2012.

### Herbivory Assays

Edge-mediated herbivory pressure on transplants was assessed using *Posidonia australis* transplants collected adjacent to the study site (Flora collection permit; DEC SOPP SW014729). Transplants consisted of two shoots and 15cm of rhizome excavated from the growing edge of a *P*. *australis* meadow. Transplants of this type are well established as being suitable for regrowth of *P*. *australis*. Each transplant used for experimentation had 2–5 intact leaves per shoot. Where leaf grazing marks or damage was observed on collected plants, the leaf was either trimmed, removed entirely or if leaf damage was extensive, the entire transplant was discarded.

To test the hypothesis that herbivory pressure on *P*. *australis* transplants is influenced by distance from the edge of the seagrass meadow, transplants were planted 2m within the meadow, on the meadow edge at 0m, and with increasing distances from the edge of the seagrass meadow into the unvegetated sand sheet (along a transect at 2m, 10m, 30m, 50m and 100m distance, Fig 1c). Four transects were sampled at 10m intervals along the edge of seagrass meadow (Fig 1c). At each distance, six replicate transplants were transplanted at 20cm spacings. Planting consisted of digging a 15cm long by 5cm deep furrow, transplants were then installed in the furrow, anchored with a wire peg and buried in sand to cover the rhizome. After 24 hours, transplants were inspected for signs of herbivory (bite marks, shredded leaves, holes). If herbivory was detected on a transplant, the transplant was collected and replaced with a new transplant in the same position. This process continued for six days to provide an estimate of the daily probability of leaf grazing.

We estimated changes in leaf area using images collected before (initial) and after (final) herbivory (Canon 30D digital SLR camera). To ensure the same leaf was used in each estimate, we oriented each transplant so that the apical shoot was on the left-hand side, and leaves were numbered consecutively from front to back (i.e. first leaf was on top, second leaf was behind etc). In this way, each leaf (2–5 per shoot) from each shoot (two per transplant) and each transplant had a unique code. A portion of this code, the transplant number, was attached to each transplant (using a wire tie and waterproof tag) when planted in the plots, to aid later leaf matching. The image was first calibrated to a standard distance of 9cm, representing a standard sized *Posidonia* leaf (at Useless Loop). Then the initial and final leaf areas were calculated using the area measurement function in the program CPCe [33].

To measure mean daily net aboveground transplant primary production we installed one additional transect with an identical number of replicate transplants (i.e. 6), plot and transplant configuration to the four replicate transects. However, in this transect we installed exclusion cages over each of the plots to prevent transplants from being removed or disturbed via grazing or bioturbation. In these plots, the net aboveground transplant primary production was measured at each distance using the hole-punch technique [34]. All six *Posidonia* transplant shoots within each of the caged plots were marked by punching a hole, with a pin, through all leaves at the top of each shoot sheath. After six days, only three to four transplants remained in each cage. We observed burrows along the sides of all the cages and fish were present within some of the cages, suggesting a caging artefact. However, surviving transplants showed no signs of grazing or physical damage and were deemed suitable for leaf growth rate measurements. Leaves from three transplants collected at each distance from the meadow edge were separated from the shoots at the top of their sheath. Once removed, the surface area of all leaves in each shoot was measured as described above. The area of new leaf growth (cm^2^ day^–1^) was defined as the distance between the initial marking scar and bottom of the leaf, plus any new unmarked leaves formed during the six day period. Growth was summed for each transplant (cm^2^ transplant^–1^ day^–1^). This value was subtracted from the change in leaf area lost to herbivores (see above) to provide an estimate of the daily change in leaf area of transplants consumed by herbivores (see equation below);

$$Growth=LeafGrowth_{\left(caged\right)}Time$$ (1)

$$GrazingRate=\left(LeafArea_{\left(I\right)}+Growth\right)-LeafArea_{\left(F\right)}$$ (2)

where Growth is mean daily leaf growth rate per *P*. *australis* transplant (cm^2^ transplant^–1^ day^–1^) grown in a cage for six days (Leaf Growth_(caged)_) multiplied by the number of days a transplant was growing *in situ* before a grazing event occurred (Time), Grazing Rate is daily leaf loss in cm^2^ transplant^–1^ day^–1^, Leaf Area_(I)_ is initial leaf area per transplant, Leaf Area_(F)_ is final leaf area per transplant (cm^2^ transplant^–1^) after grazing event.

### Transplant Survival and Establishment

We investigated longer term transplant survival and establishment of transplants with distance to the extant seagrass edge by replicating the above experimental design (excluding caged transect). We planted six replicate transplants at each distance (except -2m due to difficulty in recovering transplants within the seagrass meadow) and removed all roots from transplants using a sharp blade. This enabled accurate estimates of root development at the end of the six month experimental period without causing undue stress to the transplants [35]. After six months, the proportion of transplants remaining within plots along each transect was recorded. Net above-ground transplant primary productivity (see method description above) was measured on all surviving transplants. Transplants were harvested and root growth assessed. Roots were removed from each transplant, digitally scanned on a flat-bed scanner and analysed using WinRhizo® 4.0 software (Regents Instruments Inc., Canada) to determine root length.

### Fish Surveys

An initial fish survey was conducted in June 2012 using underwater video-cameras (2002/2005 Sony® mini DV Handicams with Carl Zeiss Wide Angle lenses). However, after the first days’ deployment it was obvious the camera equipment deterred grazers from entering the trial plots. In December 2012, we conducted a second fish survey but instead used more compact and silent solid state Go-Pro® Hero2 cameras with underwater housings. We established four new plots with fresh transplants at 10m from the extant meadow edge. Each camera was mounted on a pole and positioned near a plot corner focused on the transplants with a recording time of three hours and was installed at 8am and again at 3pm for six consecutive days.

### Statistical Analysis

Daily probability of grazing on transplants and long-term transplant survival data were tested for normality. For daily probability of grazing, each transplant was considered a replicate with distance as a fixed factor and transect as a random factor. For long term transplant survival each plot within a distance was considered a replicate (i.e. four plots with six plants in each plot) with distance as a fixed factor and transect as a random factor. A Shapiro-Wilk test revealed the data were not normally distributed for both daily probability of grazing on transplants and long-term transplant survival (R Core Team, 2014). To test for variation between transects and distances we used a fixed factor Permutational MANOVA in PERMANOVA+ (Primer e version 1.0.3). The PERMANOVA used distance as a fixed factor, and transect (1, 2, 3, 4), as a random factor. Data was transformed into a Euclidean resemblance matrix, with 9999 permutations. We found no difference in daily probability of grazing or long-term percent survival within a distance (across transects) from the meadow edge but significant differences between distances. Subsequently, to increase the power of our analyses we pooled all plants grazed within each distance for further statistical analysis. Because we were interested in the likelihood of daily grazing and long-term survival of transplants among distances, we analysed the data using logistic regression. Logistic regression provides odds ratios, with confidence intervals, which can be back transformed to provide predictive probabilities which are more easily interpreted and readily applicable than odds ratios. We applied a Bayesian inference (bayesglm, R package) with non-informative prior assumptions to the logistic regression, as proposed by [36], to obtain stable logistic regression coefficients and improve explained deviance from the mean. Following this, a Wald Chi square analysis tested for overall significance of the model. If a significant difference was detected we tested additional hypotheses about the pairwise differences in the coefficients by distance. Coefficients were then back transformed to obtain predictive probabilities. Daily leaf area loss and long term growth response (transplant leaf productivity and root length) were compared among distances using Kruskal–Wallis Chi square analysis rank sum test. This non-parametric method was used because we compared more than two independent samples (n = 6), and which had different sample sizes. Because there was no grazing at a distance of 100m, this distance was not included in the analysis of daily leaf area loss (n = 5). When a significant difference was detected we applied a HSD Tukey posterior pairwise analysis (nparcomp, R package).

## Results

### Herbivory Assay

**Fig 2:**
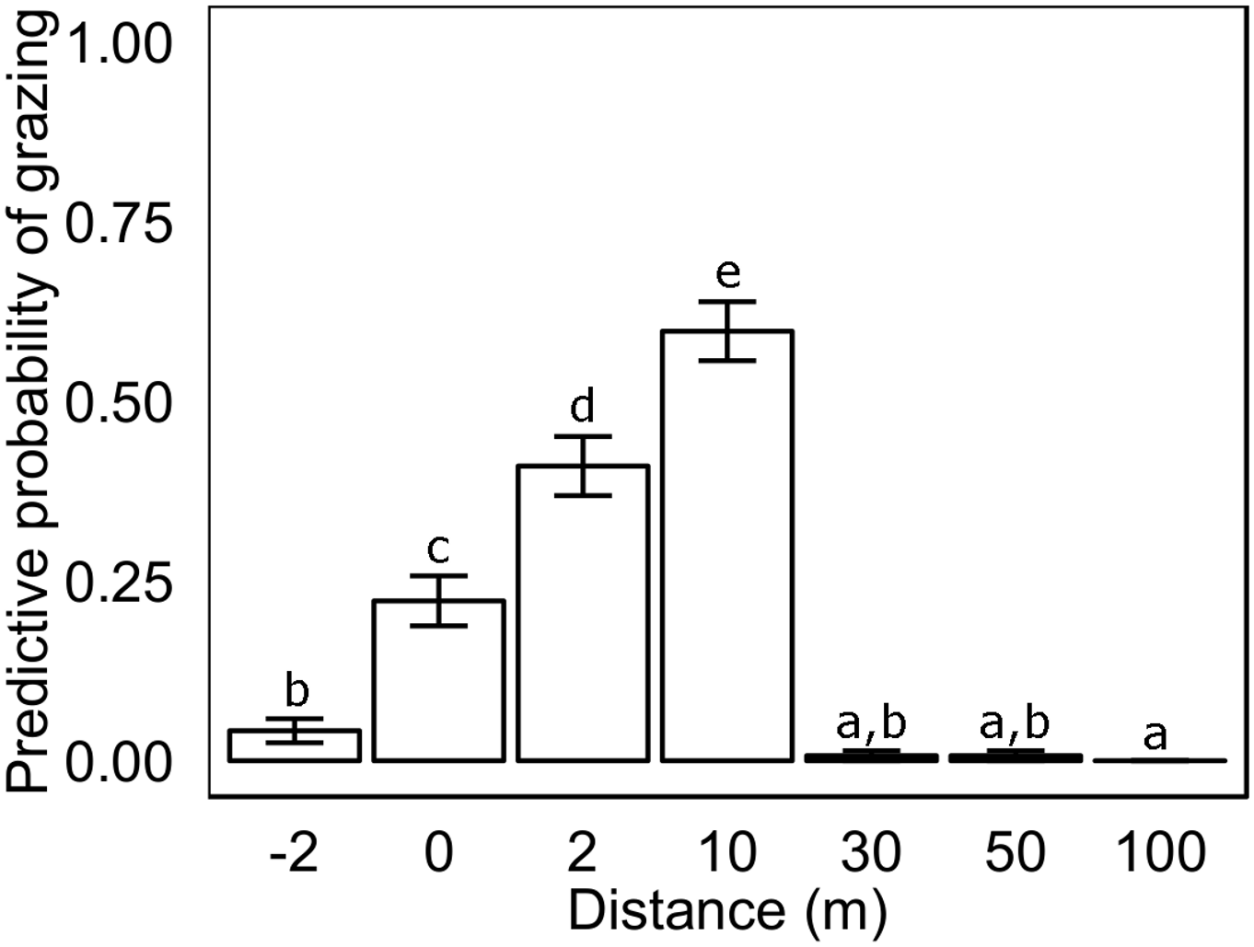
Predictive probability of daily grazing (June 2012) on *Posidonia australis* transplants planted within a *P*. *australis* seagrass meadow then at increasing distances from the *P*. *australis* meadow edge. Columns represent means ±1 SE (n = 144).

The daily probability of grazing on a *Posidonia australis* transplant was significantly influenced by distance from the edge of the seagrass meadow (x^2^_1008, 7_ = 185.6, p<0.001, Table 1). The daily probability of being grazed within the seagrass meadow (2m from seagrass edge) was low (<5%). This corresponds to observations made during the experiment of low numbers of leaf bite marks on leaves of the intact meadow at 2m, but that increased significantly to 22% on the meadow edge (0m; Table 1, Fig 2). At 2m distance into the sand sheet, the grazing risk on transplants was even greater, at ~41%, increasing to a maximum of ~60% at 10m (Table 1, Fig 2). There was a sharp decline in the probability of a transplant being grazed beyond 30m (<1%). No grazing was observed at 100m (Table 1, Fig 2).

**Table 1:** Wald Chi square analysis (and Wald chi square pairwise comparisons) for predictive probability of daily grazing and predictive probability of survival six months after planting relative to distance from the seagrass edge.

Variable			Wald Chi^2^		n	df	P(>Chi^2^)
*Probability of grazing*			185.6		1008	6	1e^-5^
*Probability of survival*			28.6		144	5	7.4e^-5^
Wald Chi^2^ Pairwise tests—*Probability of grazing*							
**Distance**	**-2**	**0**	**2**	**10**	**30**	**50**	**100**
**-2**	1						
**0**	39.4***	1					
**2**	55.7***	11.5***	1				
**10**	71.1***	39.5***	10.1**	1			
**30**	2.2	16.9***	27.5***	38.1***	1		
**50**	2.2	16.9***	27.5***	38.1***	1.9e^-30^	1	
**100**	0.05	11***	15.8***	20.5***	0.61	0.61	1
Wald Chi^2^ Pairwise tests–*Probability of survival*							
**Distance**	**0**	**2**	**10**	**30**	**50**	**100**	
**0**	1						
**2**	2.3	1					
**10**	5*	0.99	1				
**30**	10.8**	6.7**	3	1			
**50**	10.8**	6.7**	3	5.5e^-30^	1		
**100**	4.1*	0.47	0.1	4.1*	4.1*	1	

* <0.05,

** <0.01,

*** <0.001

**Fig 3:**
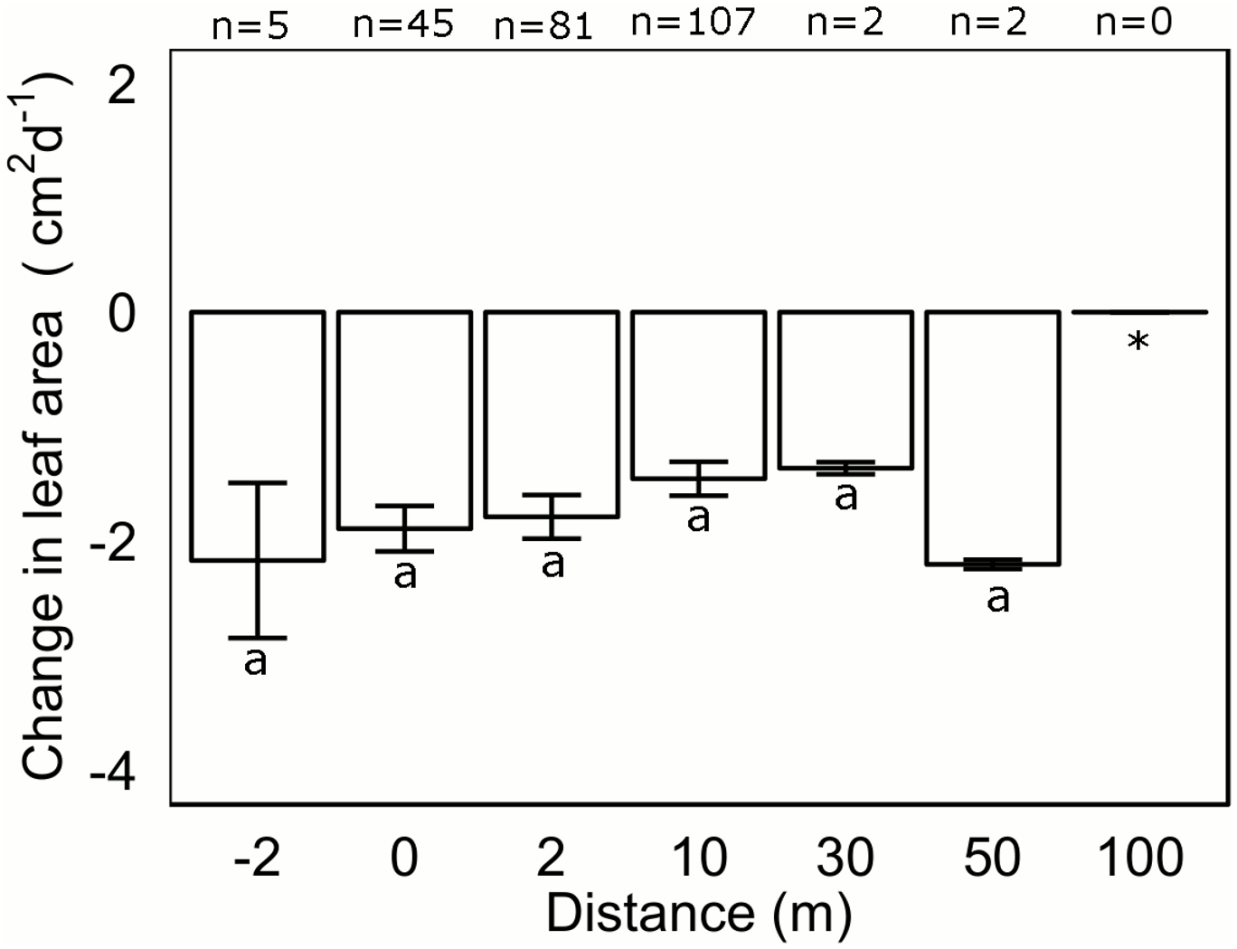
Mean change in leaf area (cm^2^ d^-1^) of *Posidonia australis* transplants (June 2012) after grazing events when planted within a *P*. *australis* seagrass meadow then at increasing distances from the *P*. *australis* meadow edge. Columns represent means ±1 SE. * not included in analysis because no grazing event was recorded at 100m.

When transplants were grazed, the daily loss of leaf area to grazing was greater than the amount of leaf production per day across all distances, except at a distance of 100m where no grazing was recorded (Fig 3). Mean leaf area lost to grazing was between 1–2 cm^2^ d^-1^ and not significantly different between distances (p > 0.05; Table 2).

**Table 2:** Kruskal-Wallis Chi square analysis (and Tukey HSD pairwise comparisons) comparing daily leaf area loss, leaf productivity (after 6 months) and root length (after 6 months) by distance from seagrass meadow edge.

Variable			K-W Chi^2^		n	df	P(>Chi^2^)
Daily leaf area loss (cm^2^ d^-1^)			6.86		242	4	0.33
Leaf productivity (cm^2^ d^-1^)			2.33		44	5	0.80
Root length (cm)			29.21		44	5	2.1e^-5^
HSD Tukey pairwise tests–*Root length*							
**Distance**	**0**	**2**	**10**	**30**	**50**	**100**	
**0**	1						
**2**	1.97	1					
**10**	2.60	0.43	1				
**30**	14.47**	14.47**	14.47**	1			
**50**	14.47**	14.47**	3.08*	-0.65	1		
**100**	1.84	-0.25	-0.59	-1.71	-1.71	1	

* = <0.05,

** = <0.001

### Transplant Survival and Establishment

**Fig 4:**
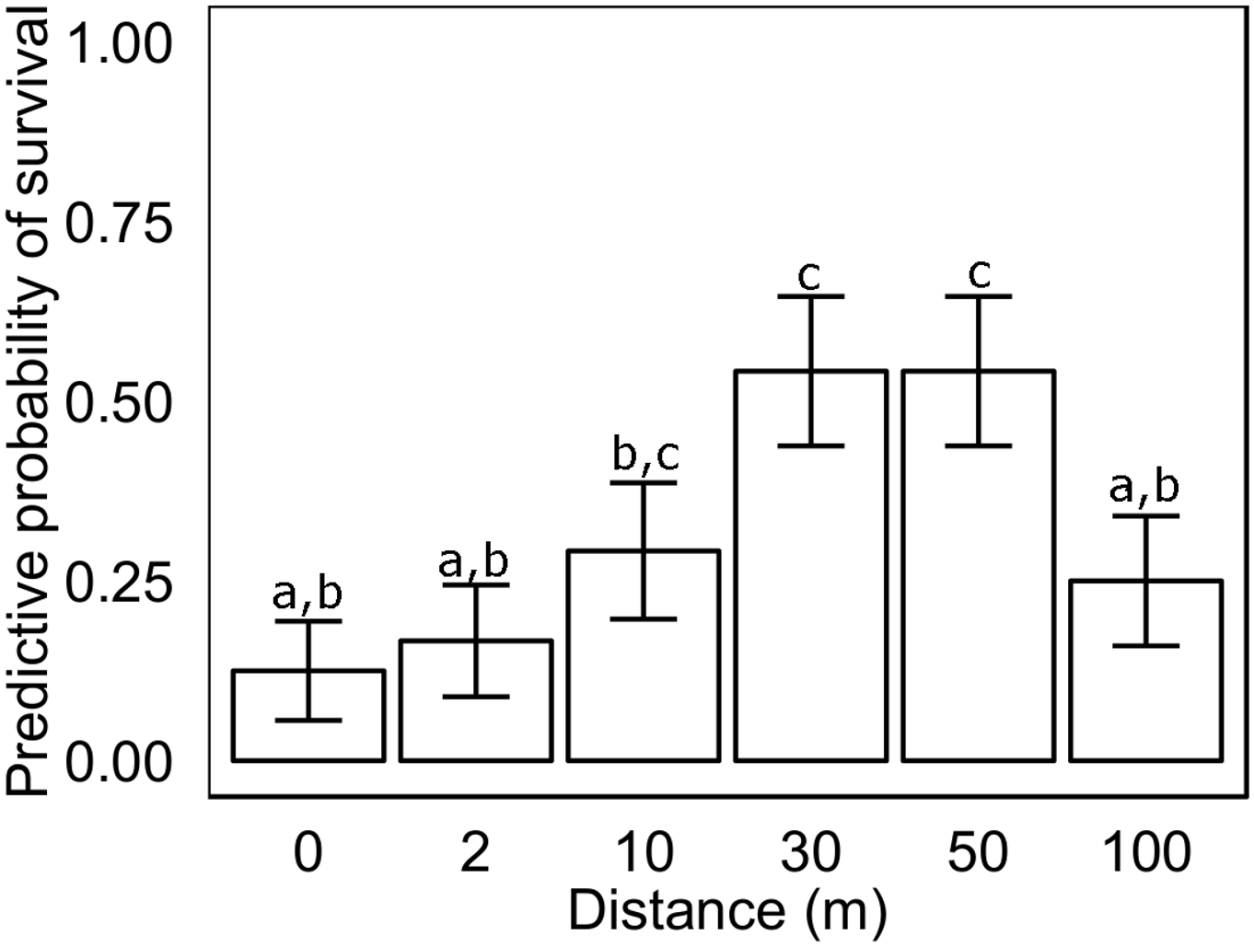
Predictive probability of survival of *Posidonia australis* transplants six months after planting (November 2012) at increasing distances from a *P*. *australis* meadow edge. Columns represent means ±1 SE (n = 4).

The probability of survival for a *Posidonia australis* transplant six months after planting was significantly influenced by distance from the edge of the extant seagrass meadow (x^2^_144, 6_ = 28.6, p<0.001, Table 1), but did not follow short-term patterns of grazing intensity. The probability of survival was lowest proximal to the extant meadow edge (<17%), and only marginally higher though not significantly different at 2m (<20%) into the sand sheet area. Transplant survival at 10m (28%), was significantly higher than at the meadow edge, but not significantly different from 2m into the sand sheet (Table 1, Fig 4). At the time the transplants were harvested we observed extensive amounts of wrack on the seafloor, consisting mainly of seagrass leaves, 10-20m inside the sand sheet but up against the extant *Posidonia* meadow. The highest probability for transplant survival (53%) was found at 30m and 50m from the extant meadow edge (Table 1, Fig 4). At 100m there was a decrease in transplant survival (25%). This decrease did not appear to be driven by grazing, since plants present at 100m had no evidence of grazing on leaves. Rather, at this distance we observed plants excavated from their furrow, most likely by bioturbators which were observed in large numbers at 100m, and included heart urchins, burrowing shrimp, and large pits presumably caused by rays or crabs.

**Fig 5:**
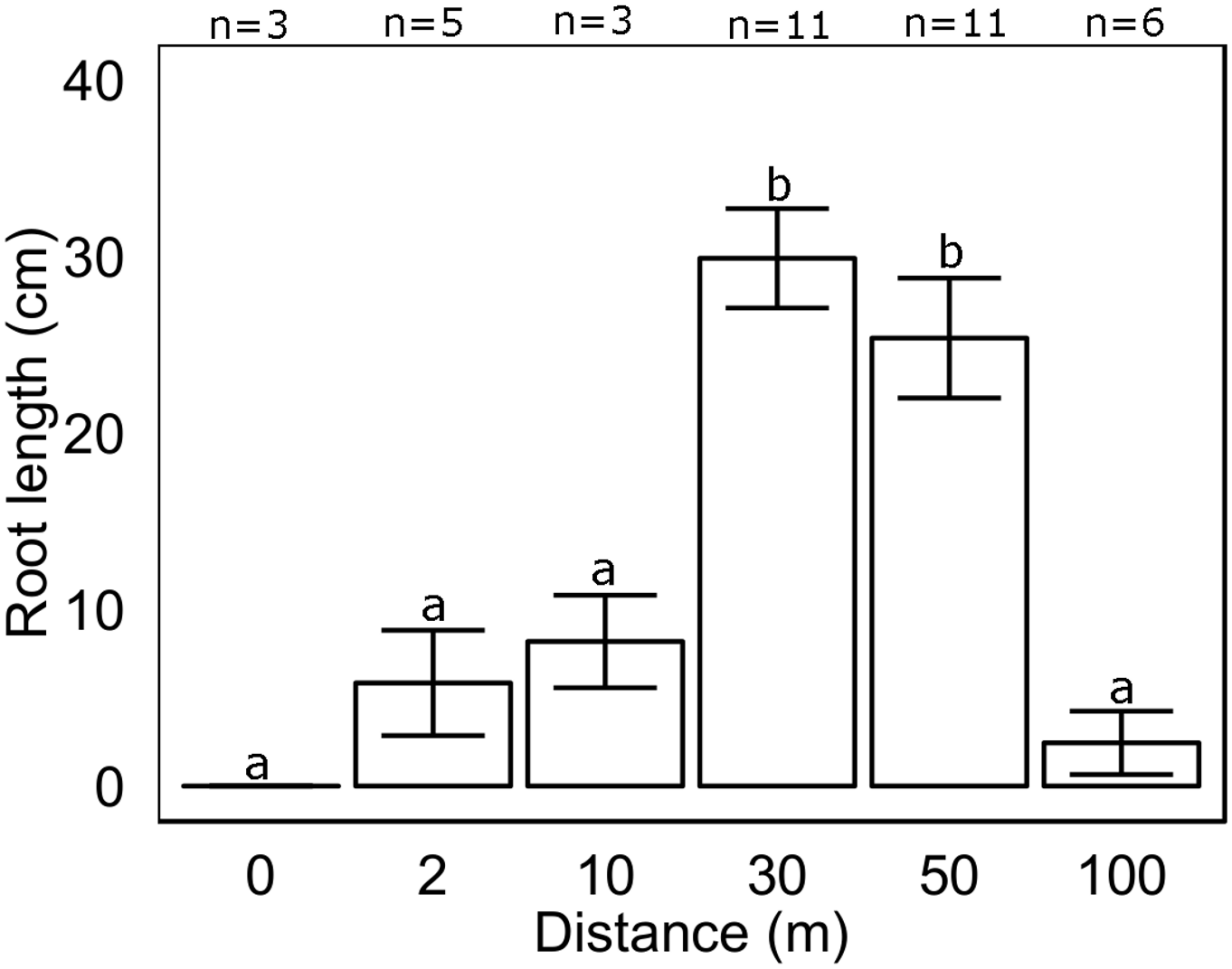
Change in root length (cm) of *Posidonia australis* transplants six months after planting (November 2012) at increasing distances from a *P*. *australis* meadow edge. Columns represent means ±1 SE.

Leaf productivity of surviving *Posidonia australis* transplants six months after planting was not significantly influenced by distance from the edge of the extant seagrass meadow (x^2^_44, 5_ = 2.33, p>0.05, Table 2). In contrast, root growth (length) changed significantly with distance from the extant meadow edge (x^2^_44, 5_ = 29.21, p<0.001, Table 2). Root lengths were more than 60% longer at 30 and 50m than at 0, 2, 10 and 100m from the extant meadow edge (Table 2, Fig 5).

### Fish Survey

Direct grazing events were not recorded on surveillance video despite evidence of grazing activity. We did however document the frequency of fish/invertebrate species observed on video (S1 Table). *Pelates octolineatus* was the most frequently observed fish species occurring in ~25% of video drops. *Kyphosus gibsoni* was also observed in video drops, though less common than *P*. *octolineatus*, and is a well-known grazer of seagrasses.

## Discussion

In a manner similar to the role that habitat margins play in other ecosystems [1,4,10], a strong plant-herbivore-grazing interaction occurs along and out from the edge of meadows of the structurally complex clonal seagrass *Posidonia australis*. The intensity of this interaction has a major effect up to 10m from the edge, but decreases sharply with distances beyond 10m. Yet the risk of grazing was minimal inside (2m) the seagrass meadow, indicating that grazers may use the seagrass meadow for refuge but are not actively grazing within it. This is surprising as the transplanted seagrass shoots are subsets of morphologically similar material within the meadow yet the herbivory focuses on the transplants rather than similar shoots within the meadow. Such selective grazing is also found in terrestrial systems where herbivory is prominent in newly transplanted greenstock yet grazing of nearby intact plants of the same species is negligible [37]

Such pronounced edge-mediated herbivory on *Posidonia* transplants could be driven by one or a combination of ecological processes. These may include; (i) *herbivore-predator avoidance or a ‘risk-aversion strategy’*—seagrass-dwelling herbivores may be willing to navigate some distance away from the safety of the more structurally complex seagrass habitat but no further in search of food [2,8,9,38–40]; (ii) *edges as foraging highways*—open areas can enhance opportunities for foraging because of navigational ease towards food [2,40,41]; (iii) *resource availability (food)*–habitat edges are often sites of new seagrass recruits and these early life-stages are typically more nutritious than adult meadows. Substantial leaf loss was also observed at the leading edge of the extant meadow (i.e. younger shoots with higher nutritional value), suggesting that this may have initially attracted herbivores to the edge; (iv) *resource availability (habitat)*—while herbivores may avoid less-preferred habitat (sand), they may still forage from bordering habitat (seagrass), therefore a gradual transition from the highest densities in the interior of the preferred habitat to the lowest densities in the interior of the adjoining habitat could be expected [19,42,43]. This transition in abundance may reflect a gradient in habitat quality that may ultimately depend on resource availability (e.g. food and shelter), but we would expect greater levels of grazing in the meadow than observed.

The daily quantity of leaf material lost to grazing was greater than the amount of leaf production per transplant per day regardless of distance from the meadow edge. However, because the probability of a grazing event (~60%) was highest at 10m from the meadow edge, we would expect transplants to lose a large proportion of their leaf material within several days of continuous grazing at this distance and therefore be impacted by leaf loss over longer timescales. Yet, we did not observe a strong pattern linking short-term grazing intensity with longer term transplant survival, suggesting herbivory was not the only factor influencing transplant survival. Some species of seagrass have the ability to compensate for leaf losses from grazing by enhancing leaf productivity [44], but this has not been demonstrated for *P*. *australis*. While we did observe evidence of leaf grazing on transplants after six months, there were no differences in leaf productivity among distances at this time. We propose that *P*. *australis* may not have the capacity for compensatory growth due to inherently slow-growth rates recorded for this species. In addition, grazing intensity in this location may be more a function of opportunistic behaviour (*see above*) rather than a persistent (i.e. over six months) threat to plants. Alternatively, intensity of grazing changed over longer timescales because of seasonal influences such as when other food sources may become available, but this requires further testing.

Although it is likely that herbivory contributed to transplant mortality, there was no clear spatial pattern as expected from an increased grazing risk at 10m, suggesting other environmental factors were also affecting survival of transplants within the study area. At a distance of 100m where grazing was negligible, we recorded greater than 70% loss of transplants after six months. Here, we observed partial or complete excavation of transplants presumably from bioturbation of sediments from mobile benthic fauna like heart urchins, rays and burrowing shrimp. At 0-20m from the extant meadow, seagrass wrack accumulated along the edge. It appeared that the edge of the extant meadow acted as a barrier to the horizontal transport of wrack preventing further movement along the seafloor, at least while we were observing the study area over six days in November. Consequently, the accumulated wrack may smother, or physically abrade transplants when it eventually moves along the seafloor. Despite the limited spatial coverage of the study area, survival differed markedly over a relatively small spatial scale of 10’s of metres. Similarly, the mechanisms we observed contributing to those spatial patterns of survival were also diverse and noticeable at such a small spatial scale.

Acute and chronic disturbances can severely impact the growth and survival of seagrass meadows [45]. *Posidonia* seagrass meadows are capable of resisting short- and even long-term disturbances by translocation of resources among physiologically integrated ramets [46]. However, individual plants may be at greater risk due to their small size and because they are physiologically independent with limited resources to compensate for such impacts. Consequently, transplants have a lower resilience to disturbance compared to physiologically integrated meadows and thus are likely to be more susceptible to physical disturbances. While we found no differences in leaf productivity of surviving plants located near the meadow edge and into unvegetated sand, root development was clearly reduced in areas where transplant survival was lowest (i.e. close to the boundary, 0–10m, and at 100m from the extant meadow). Several possibilities could account for these differences, such as conditions below ground were unsuitable for greater root development in these areas (e.g. incompatible resources such as H_2_S). Also, plants in these areas had greater levels of physical disturbance (e.g. grazing, bioturbation, smothering or abrasion from wrack) which ultimately impacted development of the root system either by disrupting the root rhizosphere or dislodging plants entirely. A third and more likely possibility, grazing of leaves reduced photosynthetic carbon fixation that resulted in more investment in maintaining leaf growth to the detriment of root growth [47,48], but is an area that requires further investigation in seagrasses. Regardless of the mechanisms, poor root development will likely have longer term negative consequences for sediment nutrient acquisition and root anchorage, and ultimately the survival of transplants.

There is little published evidence to support that grazing on seagrasses follows strong patterns in space. Edge effects between seagrass and other complex habitats (e.g. reef) have been reported [10], yet there have been few studies explicitly testing grazing intensity in relation to seagrass edges (e.g. [49]; this study). [49] found there was an increase in grazing intensity along with a reduction in grazer size with greater distances (>4m) away from the blowout edge of the sandy blowout and into the intact meadow. Although our study did not test grazing intensity at distances greater than 2m into the intact meadow, there is a substantial amount of evidence from terrestrial ecosystems indicating that edge effects can be two-sided, exerting additional constraints on the recovery or expansion of the native habitat [50]. Greater focus on non-random patterns of grazing in seagrasses is required to improve our understanding of edge effects, and other patterns in grazing.

Video did not identify grazing on our seagrass transplants however an analysis of leaf bite mark suggests that fish were the major grazers. One of the most frequently observed fish species was *Pelates octolineatus* (observed in ~25% of video drops). *P*. *octolineatus* is reported to be one of the most significant seagrass herbivores in the Shark Bay ecosystem [15]. Indirect evidence from feeding assays and gut content and fatty acid analysis revealed that seagrass forms a significant part of the diet for *P*. *octolineatus*, consuming a range of seagrass species that included *P*. *australis*. We also observed *Kyphosus gibsoni*, a known grazer of seagrasses though less common in the video observations.

## Conclusion

Seagrass edges can facilitate intense grazing compared to within meadow or unvegetated sand habitats. We found that herbivory was highly focused, operating over a small spatial scale at the edge of a large, structurally intact seagrass meadow. Because of this strong spatial pattern in daily grazing risk and substantial daily losses of leaf material due to grazing, we expected long term survival of plants to be impacted close to the meadow margin. While herbivory was a potential contributing factor to transplant survival, the relationship between short-term patterns in grazing risk and longer term transplant survival were not straightforward and suggest that other mechanisms or environmental filters were influencing longer term seagrass establishment within our study area. It is noteworthy that despite the small spatial coverage of our study area (10’s -100’s of metres) we observed strong spatial differences in grazing risk, large variation in transplant survival and root development after six months and several observed but untested mechanisms potentially influencing longer term outcomes. This clearly demonstrates that no single approach or short-term test could generate sufficient understanding of the mechanisms influencing plant establishment within this location, and that a more systematic approach is required.

## Supporting Information

S1 Table Frequency of fish and invertebrate species observations. (DOCX)
