# Supplementary material for: Edge Effects along a Seagrass Margin Result in an Increased Grazing Risk on Posidonia australis Transplants
Source: PLoS One. 2015 Oct 14;10(10):e0137778. doi: 10.1371/journal.pone.0137778 (PMC4605637; doi:10.1371/journal.pone.0137778)
Supplement: S1 Table — (DOCX) [file pone.0137778.s001.docx]

S1

| Species Name | Common Name | % Observed | Typical Activity |
| --- | --- | --- | --- |
| *Pentapodus spp.* | Butter Fish | 78.65 | Present as individuals, small or large schools (20+), Forages in sediment, seagrass wrack and on seagrass transplants |
| *Upeneus tragula* | Bar Tailed Goatfish | 44.94 | Present as individuals or small schools of 1 to 16 individuals. Forages in sediment, seagrass wrack and on seagrass transplants |
| *Pseudojuliodes elongatas* | Long Green Wrasse | 41.57 | Present as individuals or small schools 2-5 individuals, forage in seagrass wrack, near transplants and in sediments |
| *Choerodon cauteroma* | Blue Spot Tusk Fish | 39.32 | Present as individuals or small schools of 2 to 5 individuals. Forages using right pectoral fin to disturb sediment and expose food material. Also Forages in sediment, seagrass wrack and on seagrass transplants. |
| *Coris caudimacula* | Green Slender Wrasse | 25.84 | Present as individuals or large schools 100+ in Forage above seagrass canopy on suspended planktonic material, shelter in seagrass bed. |
| *Leptojulis cyanopleura* | Wrasse Weedy | 24.71 | Present as individuals or small schools 2-5 individuals, forage in seagrass wrack, near transplants and in sediments |
| *Pelates octoilineatus* | Striped Trumpeter | 24.71 | Present as individuals or in schools 20+. Forage amongst seagrass bed, wrack and sediment adjacent bed. |
| *Choerodon cyanodus* | Blue Tusk Fish | 16.85 | Present as individuals. Forage among seagrass shoots and in  wrack |
| *Plagiotremus spp.* | Blenny | 16.85 | Present as individuals or small groups, foraging in wrack & seagrass beds. |
| *Dasyatis kuhlii* | Blue Spot Ray | 10.11 | Present as individuals |
| *Sphyraena obtusata* | Striped Seapike | 8.98 | Present as individuals or large schools |
| *Paramonacanthus choirocephalus* | Hair Finned Leatherjacket | 5.61 | Present as individuals. Forage on epiphytes on the transplant leaves, yet to be seen taking a physical bite out of seagrass leaf. |
| *Sphyraena putnamiae* | Pike (Millitary Seapike) | 4.49 | Present as individuals |
| *Kyphosus Gibsoni* | Southern drummer | 3.37 | Present in Schools of 10+ individuals. Forage in seagrass bed, present grazing in first trial. |
| *Psammoperca waigeinsis* | Sand Bass | 3.37 | Present in pairs, forage among seagrass |
|  | Cuttlefish | 2.24 | Camoflagued, buried under sediment in plot. |
|  | Squid | 5.61 | Present as individuals or small groups, drifting over seagrass canopy |
| *Carcharhinus falciformes* | Silky shark | 2.24 |  |
| *Negaprion acutidens* | Lemon Shark | 3.37 | Present as individuals, seen hunting squid |
|  | Seasnake | 3.37 | Forage along seagrass boundary edge |
